# Subcapsular hepatic hematoma after extracorporeal shock wave lithotripsy for ureteral stone successfully treated with transarterial embolization: A case report

**DOI:** 10.1016/j.radcr.2026.05.008

**Published:** 2026-06-03

**Authors:** Daisuke Fujimori, Sakiko Hiraki, Julia Hamasaki, Shogo Shirane, Songhyon Cho, Fumie Sato, Yoshiaki Ichinose

**Affiliations:** Department of Radiology, National Hospital Organization Disaster Medical Center, Tokyo, Japan

**Keywords:** Extracorporeal shock wave lithotripsy, Ureteral stone, Subcapsular hepatic hematoma, Hemorrhagic ascites, Angiography, Transarterial embolization

## Abstract

Subcapsular hepatic hematoma is a rare complication of extracorporeal shock wave lithotripsy performed for ureteral stones, and reports describing severe hemorrhage requiring endovascular treatment are limited. A 45-year-old man with hypertension and obesity underwent extracorporeal shock wave lithotripsy for an asymptomatic right upper ureteral stone at a referring facility. Later that night, he developed abdominal pain and was transported to our emergency department the next day. Laboratory testing demonstrated anemia and elevated lactate. Contrast-enhanced computed tomography revealed a hepatic subcapsular hematoma along the anterior-to-inferior aspect of the right hepatic lobe, with multiple punctate enhancing nodules on the liver surface and a large volume of hemorrhagic ascites. Emergency angiography demonstrated pseudoaneurysm-like findings in distal branches supplying hepatic segments V and VI, and these lesions were treated with selective embolization using gelatin sponge particles. After embolization, his hemodynamics stabilized, hemoglobin levels did not further decline, and blood transfusion was not required. Follow-up contrast-enhanced computed tomography performed 6 days after embolization showed no contrast extravasation and decreased ascites, and the patient was discharged. Interval computed tomography confirmed gradual hematoma resolution. Clinicians should recognize subcapsular hepatic hematoma as a rare but potentially life-threatening complication after extracorporeal shock wave lithotripsy. In cases with substantial hemorrhage, prompt imaging evaluation and endovascular hemostasis can be effective.

## Introduction

Extracorporeal shock wave lithotripsy (ESWL) is an established, minimally invasive treatment for ureteral calculi and is widely used. Hematuria, renal hemorrhage, and renal subcapsular hematoma are relatively common complications of ESWL; however, subcapsular hepatic hematoma is rare and has been reported only in a limited number of cases.

We report a case of subcapsular hepatic hematoma after ESWL for ureteral stone that was successfully managed with transarterial embolization (TAE). Notably, the patient presented not only with a subcapsular hepatic hematoma but also with massive hemorrhagic ascites, reflecting a high-volume hemorrhagic presentation. We describe the clinical course and discuss the possible mechanisms with a review of the literature.

## Case presentation

A 45-year-old man (height, 160 cm; weight, 86 kg; body mass index [BMI], 33.6) had a history of hypertension treated with amlodipine. He had no known coagulopathy and was not taking any antithrombotic agents.

At another hospital, ESWL was performed for a chronic, asymptomatic right upper ureteral stone. On pre-ESWL computed tomography (CT), the stone measured 7 mm in maximal diameter with a mean attenuation of 724 Hounsfield units and was associated with mild hydronephrosis. ESWL was performed with 3,000 shock waves using a Dornier MedTech DELTA III lithotripter (Germany), with fluoroscopic targeting in the supine position and shock-wave delivery from the dorsal aspect. The procedure was performed without sedation. No incidents such as marked body movement or significant vital sign changes were reported during treatment, and the patient returned home the same day.

Later that night, the patient developed abdominal pain and was transported to our emergency department the following day. On presentation, his vital signs were as follows: heart rate, 102 beats/min; blood pressure, 100/68 mmHg; respiratory rate, 24 breaths/min; oxygen saturation, 99% on room air; and body temperature, 36.0°C. He was alert. Laboratory evaluation revealed a hemoglobin level of 8.6 g/dL, platelet count of 271 × 10^3/µL, prothrombin activity of 94%, activated partial thromboplastin time of 22.3 s, fibrinogen level of 283 mg/dL, D-dimer level of 0.5 µg/mL, and lactate level of 3.3 mmol/L, consistent with anemia and suggesting hemorrhagic shock.

Contrast-enhanced CT demonstrated a subcapsular hepatic hematoma along the anteroinferior aspect of the right hepatic lobe, with multiple small pseudoaneurysms suspected on the hepatic surface (Fig. 1). Using 3D volumetry, the estimated hematoma volume on the CT was 752 mL. A large volume of hemorrhagic ascites was also present. Given the suspected active bleeding and the large bleeding, emergent angiography and transarterial embolization (TAE) were planned. CT also revealed multiple stone fragments in the right upper ureter with an aggregate size of approximately 8 mm; compared with the pre-ESWL CT obtained at the referring hospital, the stones showed minimal fragmentation and little positional change.

**Fig. 1:**
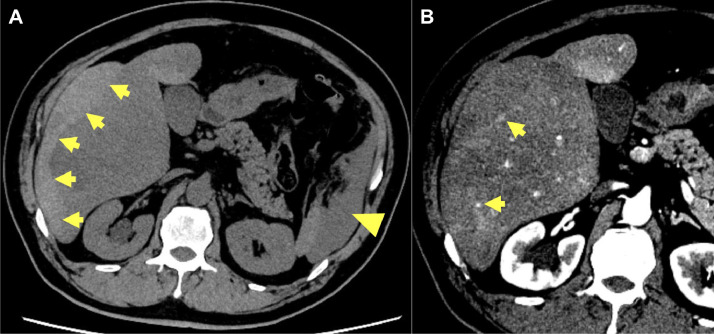
(A) Non-contrast CT demonstrates a hepatic subcapsular hematoma (arrow) and hemorrhagic ascites (arrowhead). (B) Contrast-enhanced CT demonstrates punctate enhancing nodules along the anteroinferior aspect of the right hepatic lobe (arrow).

Right common femoral arterial access was obtained, and a 4-Fr 25 cm sheath (Medikit, Tokyo, Japan) was inserted. Angiography of the right inferior phrenic artery (RIPA) was performed using a 4-Fr Shepherd hook catheter (Medikit, Tokyo, Japan). Although no definite contrast extravasation or pseudoaneurysm was identified in the RIPA, it was considered a potential contributor based on the subcapsular distribution of the hepatic hemorrhage and the expected vascular supply. A 1.9-Fr microcatheter (Progreat λ19 straight; Terumo, Tokyo, Japan) was advanced into the distal RIPA over a 0.016-inch guidewire (Meister Angled 45°; Asahi Intecc, Aichi, Japan). A gelatin sponge slurry (Serescue; Astellas Pharma, Tokyo, Japan) was prepared by pumping between two syringes through a three-way stopcock and injected. Subsequent angiography confirmed adequate flow stasis. A 4-Fr modified Simmons catheter (Medikit, Tokyo, Japan) was then advanced into the celiac artery, and celiac angiography was performed (Fig. 2A). A microcatheter was navigated into the right hepatic artery, followed by selective catheterization of two distal branches: one A5 (segment V) branch and one A6 (segment VI) branch. Selective angiography of each branch demonstrated punctate pseudoaneurysm-like lesions, which were considered the culprit lesions (Fig. 2B–C). Each branch was embolized with gelatin sponge slurry, and post-embolization angiography confirmed adequate flow stasis in all treated vessels (Fig. 2D). An additional A5 (segment V) branch without pseudoaneurysm-like findings was also embolized based on its anatomic supply and potential contribution to the subcapsular hemorrhage. These interventions achieved hemostasis. After the procedure, his hemodynamics stabilized, with no further decline in hemoglobin, and no blood transfusion was required. A follow-up contrast-enhanced CT on day 6 after embolization showed that the subcapsular hematoma volume was unchanged, hemorrhagic ascites had decreased, and no contrast extravasation was observed; therefore, the patient was discharged home. On day 30, outpatient follow-up CT demonstrated a decrease in hemorrhagic ascites. On day 76, follow-up computed tomography confirmed a marked reduction in the hepatic subcapsular hematoma, with an estimated hematoma volume of 283 mL on 3D volumetry. After discharge, the patient remained asymptomatic from the ureteral stone; therefore, no further urologic intervention was required (Fig. 3).

**Fig. 2:**
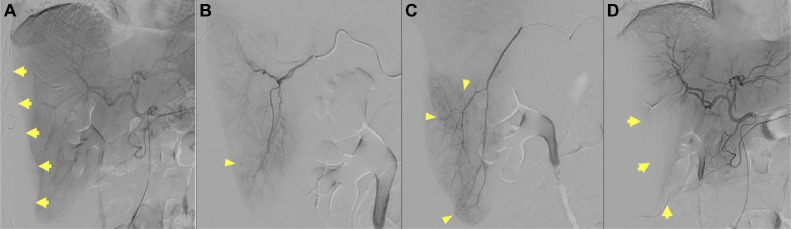
(A) Celiac artery angiography shows an avascular area corresponding to the hematoma (arrow), with leftward displacement of the right hepatic parenchyma due to mass effect. (B, C) Selective angiography from A5 (segment V hepatic arterial branch) and A6 (segment VI hepatic arterial branch) demonstrates subtle pseudoaneurysm-like findings in the distal branches (arrowhead). (D) Post-embolization common hepatic artery angiography shows adequate flow stasis in the embolized branches, with resolution of pseudoaneurysm-like findings in the anteroinferior right hepatic lobe (arrow).

**Fig. 3:**
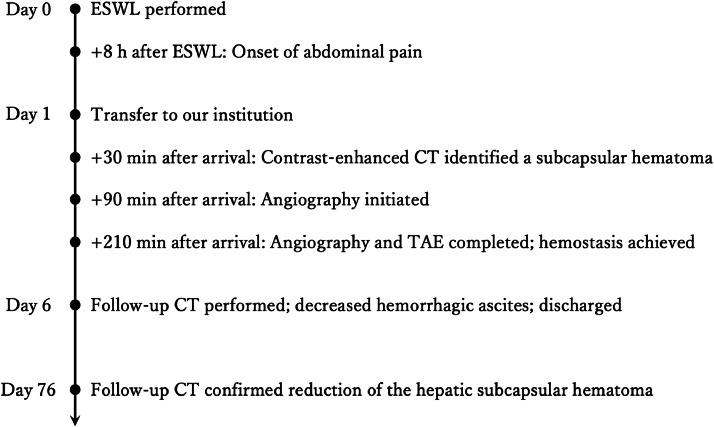
Graphical timeline of the clinical course. Day 0 indicates the day of extracorporeal shock wave lithotripsy (ESWL). CT, computed tomography; TAE, transarterial embolization.

## Discussion

Renal hemorrhage and renal subcapsular hematoma are reported complications of ESWL for ureteral stones [[1], [2], [3], [4]], whereas hepatic subcapsular hematoma is rare and has been described in only a limited number of reports [4,5]. Reported risk factors for post-ESWL renal hemorrhage include obesity, hypertension, older age, diabetes mellitus, coagulopathy, and anticoagulant use [1], and similar background factors have also been suggested as potential risks for subcapsular hepatic hematoma [5]. However, investigations addressing the specific mechanisms of subcapsular hepatic hematoma remain even more limited. In the present case, severe obesity and hypertension were present, and we considered whether additional factors might have contributed.

Skin-to-stone distance (SSD), defined as the distance from the stone to the skin measured on axial CT images, has been reported as a predictor of ESWL success (defined as stone-free status or successful fragmentation), with higher failure rates when SSD exceeds approximately 110–130 mm [6,7]. It has also been suggested that a larger SSD may predispose to off-target delivery of shock waves due to targeting difficulties and subsequent exposure of surrounding tissues, potentially increasing the risk of tissue injury [3]. In our patient, SSD was measured as 135 mm. SSD was calculated as the mean of three measurements from the stone to the skin at 0°, 45°, and 90°. Together with the poor stone fragmentation, this supports the possibility that off-target shock wave delivery occurred and contributed to the development of the hepatic subcapsular hematoma. Nevertheless, the direct association between SSD and complication rates has not been sufficiently validated.

During ESWL for ureteral calculi, respiratory motion can lead to positional changes of abdominal organs, including the ureter and liver; therefore, adjustment of the respiratory phase is important to maintain accurate targeting. Ultrasonographic observations during ESWL have shown that respiratory motion can cause a portion of shock waves to miss the stone and be delivered to adjacent tissues, which may contribute to organ injury [8]. For example, if the target is aligned to the stone during expiration but shock waves are delivered during inspiration, the liver may enter the irradiation field. When shock waves are applied to the liver, a subcapsular hematoma may occur due to the difference in acoustic impedance between the hepatic parenchyma and the liver capsule [9]. In the present case, in addition to background risk factors (hypertension and severe obesity), targeting deviation related to respiratory motion may have contributed to the development of a subcapsular hepatic hematoma and subsequent intraperitoneal hemorrhage.

Reported management options for hepatic subcapsular hematoma as the complication of ESWL include conservative therapy, TAE, and percutaneous drainage; the presence of active bleeding and concomitant infection are important factors in treatment selection [4,5,10]. In this case, given the substantial hemorrhage including hemorrhagic ascites and the suspicion of contrast extravasation on CT such that active bleeding could not be excluded, angiography and TAE were selected. After TAE, anemia did not progress and the patient was able to be discharged within a short period.

## Conclusion

We report a case of hepatic subcapsular hematoma with massive hemorrhagic ascites after ESWL for ureteral stone, which progressed to hemorrhagic shock and was successfully treated with transarterial embolization.

## Ethical approval

Ethical approval was not required for this study in accordance with local institutional policy because this is a single-patient case report.

## Patient consent

Written informed consent was obtained from the patient for publication of this case report and any accompanying images.
